# A *Piscibacillus* sp. Isolated from A Soda Lake Exhibits Anticancer Activity Against Breast Cancer MDA-MB-231 Cells

**DOI:** 10.3390/microorganisms7020034

**Published:** 2019-01-26

**Authors:** Deepesh Kumar Neelam, Akhil Agrawal, Anuj Kumar Tomer, Shreetama Bandyopadhayaya, Ankit Sharma, Medicharla V. Jagannadham, Chandi C. Mandal, Pawan K. Dadheech

**Affiliations:** 1 Department of Microbiology, School of Life Sciences, Central University of Rajasthan, Bandarsindri 305817, Ajmer, India; deepeshkphdlifesci2012@curaj.ac.in (D.K.N.); akhilagrawal@curaj.ac.in (A.A.); 2014phdmb01@curaj.ac.in (A.K.T.); 2 Department of Biochemistry, School of Life Sciences, Central University of Rajasthan, Bandarsindri 305817, Ajmer, India; 2017phdbc02@curaj.ac.in (S.B.); ankit.lifesciences@gmail.com (A.S.); ccmandal@curaj.ac.in (C.C.M.); 3 CSIR-Centre for Cellular & Molecular Biology, Hyderabad 500007, India; medicharlavj@gmail.com

**Keywords:** *Piscibacillus*, Sambhar Lake, breast cancer, anticancer activity, cell migration, stemness

## Abstract

Microorganisms thrive in extreme environments and are known for synthesizing valuable metabolites. Salt-loving microorganisms can flourish in saline environments which inhibit the growth of other microbial life, and they possess the potential to produce stable and novel biomolecules for the use in biotechnological applications, including anticancer compounds. Sambhar Lake is the largest inland soda lake in India and is an appropriate habitat for halophilic bacterial and archaeal strains in terms of diversity and potential production of bioactive compounds. In the present study, a moderately halo-alkaliphilic bacterial strain C12A1 was isolated from Sambhar Lake, located in Rajasthan, India. C12A1 was gram-positive, motile, rod-shaped, formed oval endospores, produced carotenoids, and exhibited optimal growth at 37 °C in 10–15% NaCl (pH 8). C12A1 was found to be able to hydrolyze skimmed milk, gelatin, and Tween 80 but unable to hydrolyze starch and carboxymethylcellulose. C12A1 showed 98.87% and 98.50% identity in 16S rRNA gene sequence to *P. halophilus* and *P. salipiscarius*, respectively. Nevertheless, C12A1 was clustered within the clade consisting of *P. salipiscarius* strains, but it showed a distinct lineage. Thus, C12A1 was designated as *Piscibacillus* sp. Cell proliferation assay results showed that C12A1 broth extract (BEP) decreased cell viability in breast cancer MDA-MB-231 cells, which was confirmed by the MTT (3-(4, 5-dimethylthiazol-2-yl)-2, 5-diphenyltetrazolium bromide) assay. Induction of cell toxicity was visualized by microscopy. Reverse Transcriptase PCR (RT-PCR) analysis demonstrated that BEP inhibited the expression of proliferative B-cell lymphoma-extra large (Bcl-xL) and cell cycle marker Cyclin-dependent kinase 2 (CDK2) at transcript levels. Similarly, cell migration and colony formation along with mesenchymal marker vimentin and stem cell marker BMI transcripts were found to be inhibited when cells were treated with the BEP. The anti-breast cancer potential of C12A1 indicates that microorganisms inhabiting saline-alkaline habitats, with *Piscibacillus* sp. in particular, are a promising source for discovery of novel bioactive substances.

## 1. Introduction

Diverse halophilic microorganisms inhabit hypersaline and alkaline environments such as pond brines and natural salt lakes [1]. Sambhar Lake is the largest naturally occurring inland soda (saline-alkaline) lake, situated in the state of Rajasthan, India. Its area is 230 square kilometers and surrounded by the Aravalli hills [2]. Earlier studies on Sambhar Lake indicated its halophilic bacterial diversity and a pronounced hypersalinity that was observed mainly in salterns [3,4]. Moderately halophilic bacteria have attracted more attention due to their adaptability in adverse conditions for growth. Specifically, they produce varied natural bioactive compounds such as compatible solutes, antitumor and antimicrobial substances, enzymes, polymers, and pigments [5,6].

Halophilic bacteria such as *Halobacterium* sp.TM, *Salinibacter*, *Halobacillus halophilus*, *Pseudomonas halophila* and *Haloferax mediterranei* have been isolated from varied habitat [7,8,9,10,11]. These halophilic strains are known to produce potential natural bioactive compounds that maximize the efficacy and minimize the toxicity or side effects of cancer treatment. Cancer is one of the leading causes of mortality and morbidity all over the world, resulting in a large economic burden on the world population [12]. Breast cancer is one of the most common cancers in the world including countries such as India [13]. In fact, 90% of cancer patients die due to the metastasis of cancer [14]. Earlier studies on anticancer activity of bioactive compounds from halophilic bacteria were focused on cancer cell types other than breast cancer cells. Out of 45 moderately halophilic bacterial strains isolated from sediment and saline water from the Weihai Solar Saltern China, 5 strains such as whb45 (*Halobacillus trueperi*), whb43 (*Halomonas sp*.), whb36 and whb3 (*Halomonas ventosae*), and whb33 (*Halomonas salina*) showed remarkable cytotoxic activity with favorable IC_50_ value against tumor cells Bel 7402 (Hepatocellular carcinoma) [15]. Marine bacteria are well known for producing new anticancer compounds such as Ɛ-Poly-L-lysine (Ɛ-PL). An antimicrobial compound which possesses anticancer activity was extracted from *Bacillus subtilis* SDNS [16] and showed highest inhibition against HeLa cell as compared to HepG2 and CaCo cell lines. Similarly, pelagiomicin A, isolated from marine bacterium *Plagiobacter variabilis* exhibited antitumor activity against HeLa, BALB3T3 and BALB3T3/H-ras with the IC_50_ values at concentrations 0.04, 0.02 and 0.07 µg/mL, respectively [17]. Other bioactive molecules which have attracted much interest were biosurfactants of *Halomonas* sp., BS4 and levan (polysaccharide). These isolated molecules suppressed the cell viability in mammary epithelial carcinoma cell lines and human breast cancer MCF-7 cells [18,19]. To search for new antitumor compounds, a novel phenazine derivative together with six known compounds isolated from *Bacillus* sp. exhibited cytotoxicity against P388 and K562 cell lines [20]. This demonstrated the possibility of producing an eco-friendly drug from extremophilic organisms [18]. Furthermore, levan a (2→6)-d-fructan polysaccharide produced by *Halomonas smyrnensis* AAD6^T^ exhibited time- and concentration-dependent anticancer activity on human breast cancer MCF-7 cells [19]. Similarly, the cytotoxic effect of moderately halophilic bacterial bioactive compounds was observed against different tumor cells, namely the Bel 7402 [21] and HeLa S3 cell lines [16], human umbilical vein endothelial cells (HUVEC) and adipose-derived mesenchymal stem cells (MSCs) [22]. It was found that halophilic bacteria possess distinctive metabolic and physiological capabilities [16]. 

Identification and characterization of culturable microbial communities from saline lakes will not only fortify available databases of halophilic microbes for basic research but also benefit our knowledge regarding their potential use in various fields [23]. Sambhar Lake is the largest inland saline-alkaline lake which is in the northwest part of India. Considering the vastness of Sambhar Lake and an appropriate habitat of halophilic bacterial and archaeal strains in terms of diversity and potential production of bioactive compounds, the present study was undertaken to isolate and characterize strains from Sambhar Lake (a soda lake) to determine if any exhibit anti-breast cancer activity. In this study, we successfully purified C12A1, belonging to *Piscibacillus* sp., and investigated its anticancer activity against the MDA-MB-21 breast cancer cell line.

## 2. Materials and Methods

### 2.1. Sampling and Isolation of Bacterial Strain

The Sambhar Lake is the largest inland saline-alkaline lake in India. The lake lies within the latitudes of 26°52′–27°02′ N and 74°54′–75°14′ E [23]. Water samples were collected in the summer season on 28 May 2015 from opposite side of sludge gate Guda Jhapok dam area of the lake. Samples were obtained up to 15 cm in depth and stored in sterile bottles (1 liter and 125 ml glass or polypropylene). Samples were transported to the laboratory on ice where they were processed within 3 hours post-collection and stored at 4 °C until analysis. The physicochemical conditions such as pH and temperature were determined at the sampling site by multi-parameter PCSTestr 35 (Eutech instrument, Oakton, Singapore). Other physicochemical parameters such as salinity, conductivity and total dissolved solids were determined in the laboratory with the help of deluxe water and soil analyzer kit, model-172 (Electronics India, India).

Water samples were initially inoculated for enrichment in the modified C broth medium [24] containing (g/L) NaCl, 120; KCl, 6.0; MgSO_4_·7H_2_O, 29.0; MgCl_2_·6H_2_O, 19.5; CaCl_2_·2H_2_O, 1.1; NaBr, 0.8; NaHCO_3,_ 0.2; and Yeast extract, 5.0. The pH of the medium was adjusted with NaOH (to pH 8.0) and the sample was incubated at 37 °C on a rotatory shaker (120 rpm) for 7 days. After incubation, a single colony was isolated by sample serially diluting and spread on agar containing C modified medium. Resultant growing colonies were subsequently purified by repeatedly streaking on agar containing C modified medium in petri plates. The isolated strain was designated as C12A1 and kept at 4 ºC and sub-cultured at 15-day intervals for further use.

### 2.2. Morphological Studies of the Isolated Strain

Cellular morphological characteristics (cell form, cell size, cell arrangement and spore production) were determined by Axio Lab.A1 (Carl Zeiss, Germany) microscope from cultures grown on C modified medium at 37 °C for 7 days. Gram staining was performed using standard microbiological techniques [25].

### 2.3. Metabolic and Growth Characteristics

The effect of the temperature on growth of C12A1 was examined by varying range of temperature from 5 to 55 °C in increments of 5 °C in C modified medium with continuous shaking on a rotatory shaker (150 rpm). Determination of the effect of pH on the growth of C12A1 was observed in C modified medium by adjusting the pH values to 5, 6, 7, 8, 9, 10, 11, and 12 using Tris-HCl, sodium hydroxide and glycine/sodium hydroxide buffers [26]. The growth at different salt concentrations was determined in various salinity ranges from 0–30%. The growth was observed based on the turbidity at OD_600_ by using spectrophotometer (Halo DB-30 UV-Visible Double Beam, Dynamica, Singapore). All physicochemical experiments were done in triplicate and run for 7 days [27].

Antibiotic susceptibility tests were performed by spreading the suspension of C12A1 on agar plates containing C modified medium and incubated at 37 °C for 7 days. Antibiotic discs (Himedia) of chloramphenicol (7 µg), gentamycin (50 µg), streptomycin (25 µg), kanamycin (5 µg) ampicillin (10 µg), penicillin G (1 unit), tetracycline (25 µg) and sulphatriad (300 µg) were applied aseptically on spread culture. Sensitivity towards different antibiotics was measured based on the presence of a growth inhibition zone [27,28].

### 2.4. Biochemical Characteristics and Enzyme Assays of the Isolated Strain

Biochemical characterization of C12A1 was performed by standard tests such as indole production, catalase activity, MR-VP test, urease activity, nitrate reduction, acid production from different carbohydrates [29]; oxidase activity, and citrate use [30]. All biochemical experiments were performed at 37 °C for 7 days. The ability to hydrolyze starch [31], skimmed milk [26], gelatin [29], carboxymethylcellulose [32] and Tween 80 [33] was assessed after growth on agar containing the C modified medium. The test for gelatin was performed in liquid medium as previously described [29]. All experiments were performed in triplicate.

### 2.5. Pigments Analysis

The pigment was extracted according to the method described previously [34] with some modifications. Briefly, 70 ml of C modified broth cultures were centrifuged at 9000 g for 10 min at 4 °C. The supernatant was discarded, and cold methanol was added to the pellet. Cells were homogenized using a micro pestle to facilitate extraction and followed by centrifugation at 9000 g for 7 min at 4 °C. Successive extractions were carried out until cells became colorless. The extract was wrapped in aluminum foil to protect it from light and stored at 4 °C. The UV-Vis spectrum of extracted pigment was recorded at 200–700 nm using a spectrophotometer (Halo DB-30 UV-Visible Double Beam, Dynamica, Singapore).

### 2.6. Extraction of Genomic DNA 

Genomic DNA of halophilic bacterial strain C12A1 was extracted using the earlier described protocol [35] with some modifications. Briefly, bacterial biomass was pelleted at 9000 g for 5 min and after that, 500 μL of lysis solution was added and samples were incubated at 65 °C for 5 minutes in a water bath. The genomic DNA extraction was carried out in three steps using different proportions of organic solvents. An equal amount of Chloroform:Isoamyl alcohol (24:1) was added and gently emulsified by inversion 3–5 times and centrifuged at 9000 g for 5 min. Aqueous phase was separated into a sterile centrifuge tube and again extracted with the equal volume of Phenol:Chloroform:Isoamyl alcohol (25:24:1) at 9000 g for 5 minutes. The separated aqueous phase was transferred into the sterile centrifuge tube and extracted with equal amount of Chloroform:Isoamyl alcohol (24:1) and centrifuged at 9000 g for 5 minutes. The upper aqueous phase containing DNA was transferred to a new tube and an equal amount of Propan-2-OL (iso-propyl alcohol) was added, and then the sample was mixed gently by inversion at room temperature and centrifuged at 9000 g for 3 minutes. The supernatant was removed completely, and the DNA pellet was dissolved in 70 μL of NaCl (1.2 M) solution. Genomic DNA was precipitated by adding 300 μL absolute cold ethanol and aliquots were incubated at −20 °C for 7 min. The ethanol was discarded after centrifugation at 9000 g for 5 minutes. The pellet was washed once with 70% cold ethanol and centrifuged at 9000 g for 5 minutes. The pellet was then dissolved in 50 μL deionized water by gentle vortexing and kept stored at −80 °C until further use. 

### 2.7. Amplification of 16S rRNA Gene and Phylogenetic Analysis

The 16S rRNA gene from genomic DNA was amplified using the universal 27F forward [36] and 1495R reverse primers [37]. The PCR thermal cycling conditions were as follows: hot start at 98 °C for 5 min, followed by 35 cycles of denaturation at 95 °C for 30 s, annealing at 56 °C for 45 s, elongation at 72 °C for 90 s and a final extension at 72 °C for 5 min. The PCR product was purified with GeneJET PCR purification kit (Thermo Fisher) as per the manufacturer’s protocol. The partial sequence of 16S rRNA gene was obtained by sequencing of amplified DNA of both strands. The partial sequence of 16S rRNA (1415 bp) has been submitted to GenBank (Accession no. MH636303).

Initially, a similarity search was performed using the ‘Identify’ option of the EzTaxon http://www.eztaxon.org and RDP (https://rdpNaNe.msu.edu) databases. The closest sequences were retrieved, and percentage similarity was checked after manual alignment using MEGA 7 [38]. Other sequences used for phylogenetic tree construction were retrieved from GenBank (NCBI database). Sequences of the 16S rRNA gene used for phylogenetic analysis were retrieved from the nucleotide database of NCBI using BLASTN (www.ncbi.nlm.nih.gov/blastn) and aligned with the sequence of C12A1 using MUSCLE software [39] built into MEGA 7. Alignment was refined using the Manual Sequence Alignment Editor, Align v05/2008 [40] and the same software was used to calculate sequence similarity (identity matrix) of the closest sequences considering all positions of the alignment including gaps. For the phylogenetic analyses, primarily a large group of sequences (>1200 bp) was taken into consideration, and later a smaller subset was created based on sequence relatedness by excluding strains of uncertain affiliation. A phylogenetic tree was constructed by the maximum-likelihood (ML) method using MEGA 7 with default settings, employing a K2+G+I model of nucleotide substitution, which was the best fit to the presented dataset according to the Bayesian Information Criterion (BIC). Bootstrapping of 1000 replication was calculated to find out confidence values for the edges of the best scoring ML tree. Moreover, maximum parsimony and neighbor joining trees were also constructed using MEGA 7 and the topologies of all three trees were compared to validate the phylogenetic position of C12A1 strain (data not shown). *Planococcus citreus* (X62172) was chosen as the outgroup for building a phylogenetic tree.

### 2.8. Extraction of Metabolites

C12A1 was grown in C modified medium at 37 °C for 7 days at 150 rpm. The broth containing bacterial culture was centrifuged at 9000 g for 15 min at 4 °C to remove the biomass and cell debris. The supernatant was extracted with ethyl acetate 1:1 (*v*/*v*) [41]. The organic phase was separated and condensed in a rotary evaporated with vacuum (BT 351 Rotary Evaporator, BIOTHEC, Germany). The condensed broth extract of *Piscibacillus* sp. C12A1 (BEP) was dried in a desiccator and stored at 4 °C.

### 2.9. MTT Assay 

Anticancer activity of C12A1 broth extract was evaluated by MTT (3-(4,5-dimethylthiazolzyl-2-yl)-2, 5-diphenyltetrazolium bromide) assay as previously described [42,43]. Human breast cancer cells (MDA-MB-231) were cultured in Dulbecco’s Modified Eagle Medium (DMEM), supplemented with 10% fetal bovine serum, 1% penicillin/streptomycin at 37 °C in a humidified atmosphere of 5% CO_2_. The MDA-MB-231 cells (5 × 10^3^ cells/well) were seeded in 96-well plate for 24 h. The cells were incubated with different concentrations of BEP (62.5, 125, 250 and 625 µg/mL) for 24 h. The cell viability was measured using an MTT assay; 10 µl of MTT solution (5mg/ml) was added to each well of 96 well plate, then cells were incubated at 37 °C for 2 h, and then media was completely removed. DMSO (Dimethyl sulfoxide) was added to dissolve the stain, and optical density was measured at 530 nm on an ELISA (Enzyme-linked immunosorbent assay) plate reader. Data represented the average values of three independent measurements. IC_50_ (inhibitory concentration at median level) value was defined as the concentration of BEP at which there was 50% growth inhibition of the cells [15,44].

### 2.10. RNA Isolation and RT-PCR Analysis from MDA-MB-231 Cells

Total RNA from MDA-MB-231 cells was extracted by TRIzol reagent as described before [43,44,45]. MDA-MB-231 breast cancer cells were plated in 35 mm plates and were maintained in DMEM supplemented with 10% FBS and 1% pen-strep at 5% CO_2_ at 37 °C. The seeded cells were then grown until they reached 90–100% confluency. After reaching confluency, plates were treated with the crude extract of C12A1 (125 µg/mL) for 24 h. The cells were harvested, and the total RNA was extracted using the TRIzol reagent. Furthermore, the cDNA was synthesized using 1µg of the total RNA isolated using the Verso cDNA kit (Thermo Scientific, Lithuania, EU). For carrying out the semi-quantitative PCR, 1 μL of cDNA was used along with the gene specific primers in a Proflex PCR system (Applied Biosystem by Life Technologies, Singapore). The primers used in the PCR are given in Table S1.

### 2.11. Scratch Assay for Cell Migration

To determine the cell migration, a scratch assay was performed as described before [44,46,47]. In brief, the cells were plated in 35 mm plates and allowed to grow until they reached 95–100% confluency. Later, the MDA-MB-231 cells were exposed to 125 µg/mL of C12A1 crude extract for 24 h, while normal DMEM media was used as the control. To avoid non-specific cytotoxicity (data not shown), crude extract with a lower dose was used in this assay. The experiment was done in triplicate. Cell migration pictures were captured using an inverted microscope (Carl Zeiss, Germany). The area of the migration was calculated using the Image J software and the values were statistically analyzed using Graph Pad Prism software.

### 2.12. Colony Formation Assay for Stemness

To determine the stemness degree of MDA-MB-231, cells were plated in a seeding density of 2000 cells/ well in 6 well plates as described before [44]. The cells were then treated with C12A1 crude extract (62.5 µg/mL) in separate wells and incubated for 5 consecutive days. Crude extract with a lower dose was used in this assay, as the experiments were seeded at a lower cell density and were incubated for an extended time. After five days, formed colonies were fixed by ice-cold methanol and later stained with crystal violet. The experiment was done in triplicate. Photographs of the stained plate and the colonies were captured by means of a camera and a bright field inverted microscope (Carl Zeiss, Germany) respectively.

### 2.13. Statistical Analysis

The mean values obtained in the treatment and control groups were compared and significant differences were determined by one-way ANOVA. Student t test (Graphpad prism) was used to find the statistical differences between control and treated groups. Here, *p* < 0.05 was considered as statistically significant.

## 3. Results

### 3.1. Physicochemical Properties Sampling Site

At the time of sampling, the pH and temperature of water were 9.42 and 40 °C, respectively. The salinity was calculated to be 5.18%. Furthermore, conductivity was 18.41 ms and total dissolved solids were estimated to be 16.92‰.

### 3.2. Morphological, Physiological, and Biochemical Characterization of the Isolated Strain

**Figure 1:**
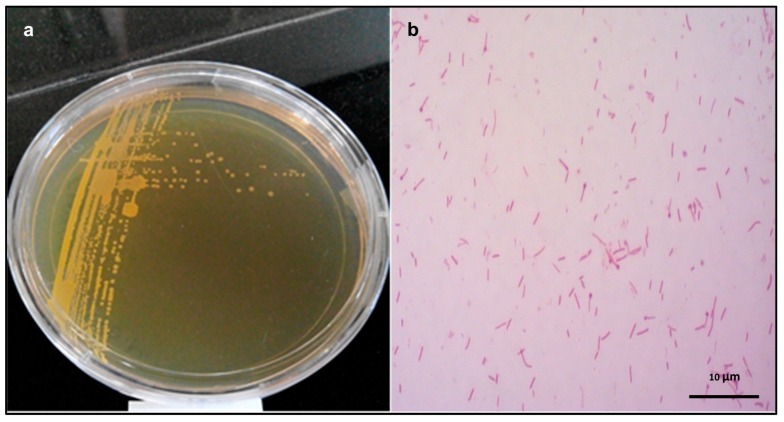
(**a**) Colonies of the *Piscibacillus* sp. C12A1 on C modified medium. (**b**) Bright field microscopy showing C12A1, gram-positive rod with oval endospores.

The colonies of isolated halo-alkaliphilic bacterium strain C12A1 were circular in shape, convex, translucent-smooth and cream to yellow in color (Figure 1a).

**Figure 2:**
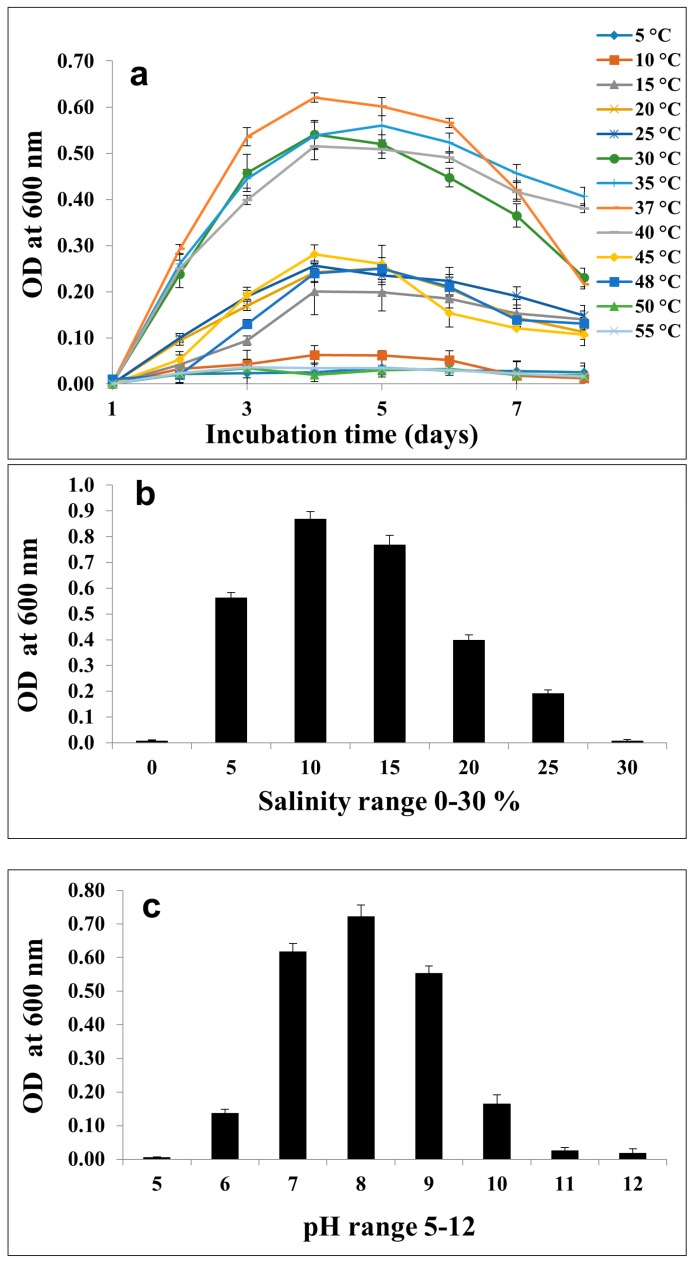
Growth pattern of the strain C12A1. (**a**) Temperature 5–55 °C. (**b**) 0–30% NaCl concentration in C modified medium. (**c**) pH ranges 5–12.

C12A1 was gram-positive, 0.3–0.5 × 1.5–3.0 µm in size, motile, rod-shaped, and oval endospore forming (Figure 1b). Growth was observed from 15 to 50 °C with optimum growth at 37 °C (Figure 2a). Optimal growth was achieved in 10–15% NaCl and no growth observed in the absence of NaCl (Figure 2b). In terms of growth based on pH, C12A1 exhibited growth between pH 6–10, with optimum growth at pH 8 (Figure 2c).

C12A1 was characterized using various conventional biochemical tests (Table 1). The strain showed a positive result for catalase and oxidase but negative for urease production.

**Figure 3:**
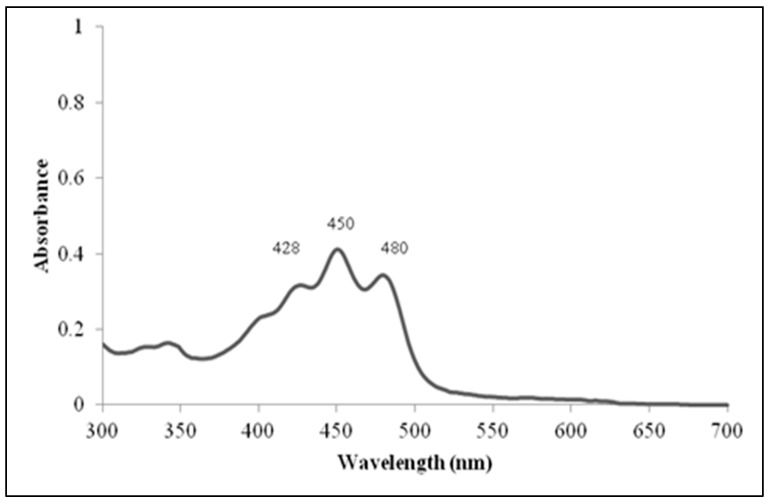
UV-visible spectrum of yellow pigment extracted in methanol showing carotenoids peaks at 428 nm, 450 nm, and 480 nm.

C12A1 gave a negative result for indole and H_2_S production and a negative result in citrate use. Acid was not produced in the presence of the following carbohydrates: D-glucose, D-maltose, sucrose, D-fructose, glycerol, and D-xylose. Methyl red, Voges-Proskauer test was negative, and the isolate gave a positive result for nitrate reduction. C12A1 was able to synthesize proteases when exposed to skimmed milk and gelatin as assessed using a plate assay for exoenzymes production. Furthermore, C12A1 displayed a positive test with Tween-80, indicating its potential to produce lipases. Amylase and cellulose production was not observed with starch, nor with carboxymethylcellulose. The antibiotic sensitivity tests showed that C12A1 was sensitive to chloramphenicol, gentamycin, ampicillin, penicillin G, sulphatriad, and tetracycline, with resistance to kanamycin and streptomycin (Table 1). The pigment of the methanolic extract was yellow and the absorption spectrum showed three peaks at 428 nm, 450 nm, and 480 nm (Figure 3).

**Figure 4:**
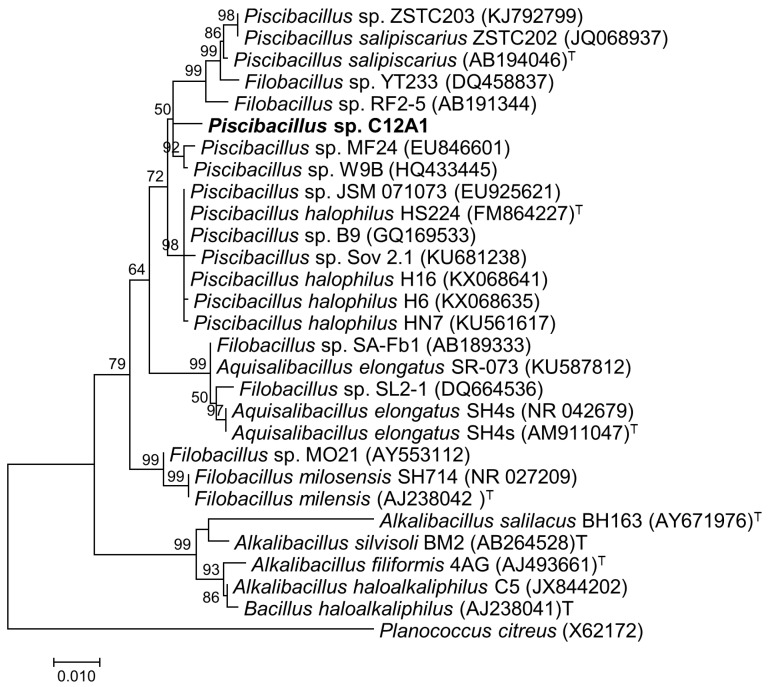
Phylogenetic tree showing the relationships between the strain C12A1 and closely related bacterial species based on 16S rRNA gene sequences. The phylogenetic tree was generated by the maximum-likelihood method and numbers above branches indicate bootstrap support data (>50%) obtained from 1000 replicates. The strain investigated in the present study is in larger bold font. ^T^ indicates type strain.

Phylogenetic analysis based on 16S rRNA gene sequencing comparisons revealed that C12A1 was close to the genera *Aquisalibacillus*, *Filobacillus*, and *Alkalibacillus*. C12A1 clustered within a clade comprising members of genus *Piscibacillus* (Figure 4). This clade was divided into two sub-clades and each one consisted of strains related to *P. salipiscarius* and *P. halophilus*. The clade of *Piscibacillus* in which all strains of *Piscibacillus* clustered was supported by 72% bootstrap. However, the sub-clade consisting of *P. salipiscarius* strains was supported by only 50% bootstrap and the distinct branch of C12A1 showed bootstrap values less than 50. Most of the sequences included in the clade of *Piscibacillus* have not yet been published. These strains were obtained across varying habitats of the Asian continent as mentioned in the nucleotide database of NCBI (http://www.ncbi.nlm.nih.gov).

### 3.3. Effect BEP on Breast Cancer Cells

**Figure 5:**
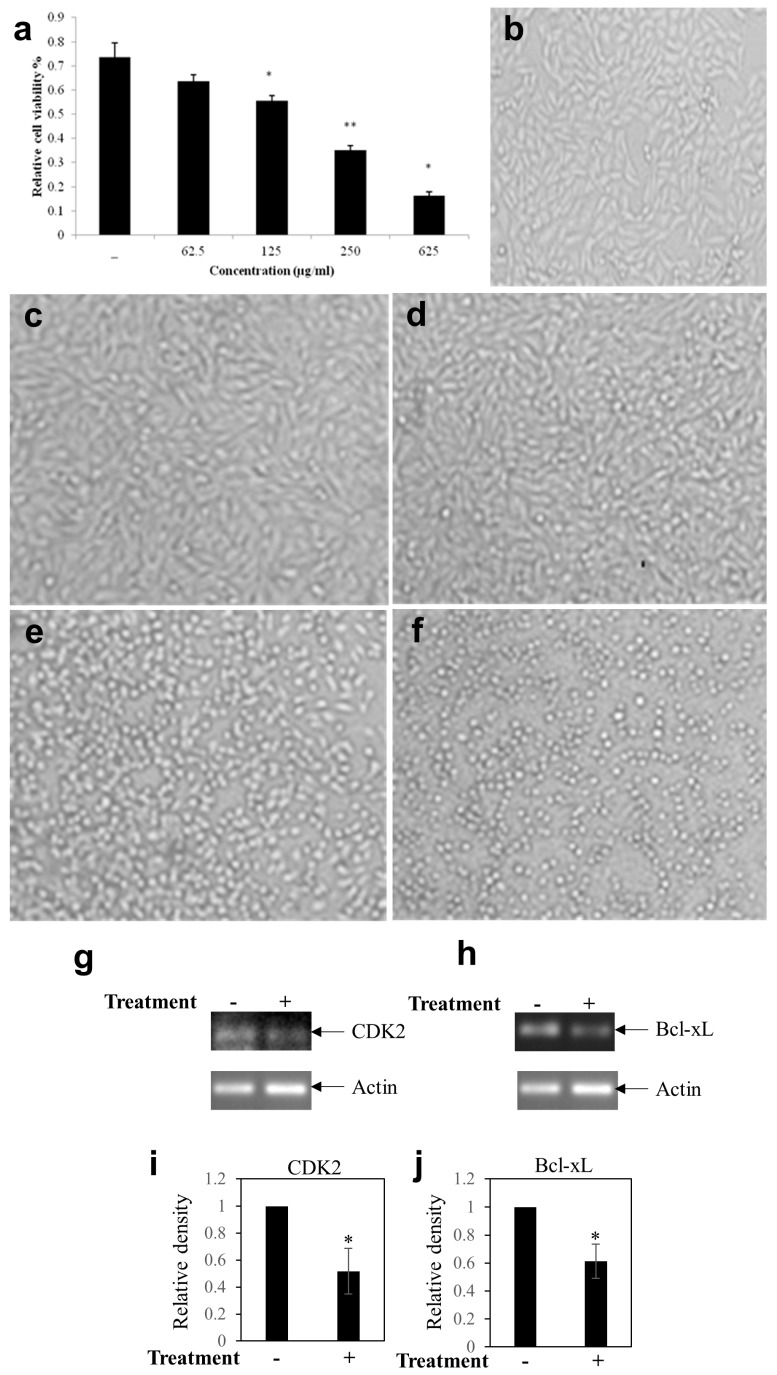
Effect of BEP (Broth extract of *Piscibacillus* sp.) on MDA-MB-231 cell viability and cell cycle associated genes of breast cancer cell (**a**) Effect of various concentrations (as shown in graph) of BEP on MDA-MB-231 cell viability. Results are the mean of three independent measurements. Significant differences were determined by one-way ANOVA, * *p* < 0.05 and **p < 0.01 vs control. (**b**–**f**) Images of MDA-MB-231 cells morphology after treatment with increasing concentration (0, 62.5, 125, 250 and 625 µg/mL) of BEP. RT-PCR analysis from the total RNA extracted from BEP treated and untreated cells (**g**) CDK2 gene and (**h**) anti-apoptotic Bcl-xL gene. Bars (**i**,**j**) showed the densitometry analysis (ratio of concerned gene/Actin) of the bands for CDK2 (**i**) and Bcl-xL (**j**). Reported values represent the mean ± SEM of three replicates. * *p* < 0.05 vs. control. The expression of CDK2 and Bcl-xL was found to be downregulated in the treatment as compared to the control. Actin was considered as the internal loading control. The expression of CDK2 and Bcl-xL were found to be downregulated in the treatment as compared to the control.

To determine the anticancer potential of the BEP, MTT cell viability assay was performed on the metastatic breast cancer cell line, MDA-MB-231. The MDA-MB-231cells were treated with increasing concentrations of BEP (62.5 to 625 µg/mL) for 24 h. BEP significantly inhibited cell viability in a dependent manner. (Figure 5a).

The IC_50_ value of crude extract was observed at 186.73 µg/mL concentration. Moreover, microscopic visualization indicated the cytotoxicity of BEP on cancer cells (Figure 5b–f). RT-PCR analysis documented that the crude extract inhibited the anti-apoptotic gene Bcl-xL and cell cycle promoting gene CDK2 transcript levels (Figure 5g–i). Taken together, these data indicate that BEP has anticancer potential.

### 3.4. Effect of BEP on Cell Migration and Epithelial to Mesenchymal Transition of MDA-MB-231 Cells

**Figure 6:**
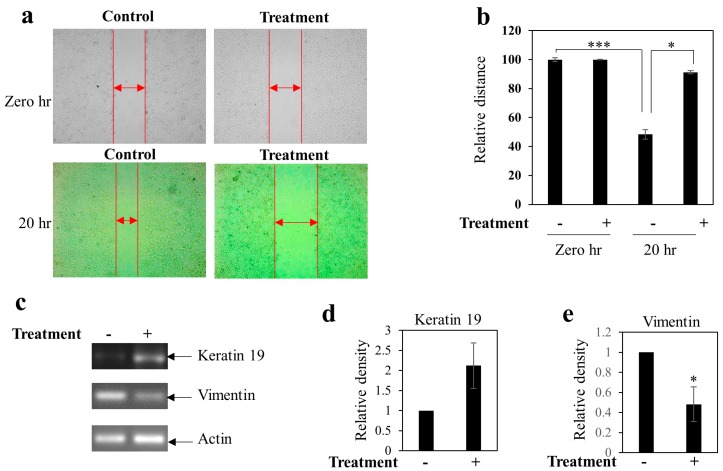
BEP inhibited cellular migration in the metastatic breast cancer cell MDA-MB-231 (**a**) Representative images at baseline and after 20 hours of treatment with BEP (**b**) The bar graph represents the relative distance that needs to be travelled by the cells to fill the gap created by the scratch. Values represent mean ± SEM of triplicate measurements. Significant differences as determined by one-way ANOVA, *** *p* < 0.001 vs. control at 0 h, and * *p* < 0.05 vs. control at 20 h. (**c**) Effect of BEP on the expression of the genes associated with the epithelial to mesenchymal transition (EMT) markers associated with breast cancer. Bars (**d**,**e**) showed the densitometry analysis (ratio of concerned gene/Actin) of the bands for Keratin 19 (**d**) and Vimentin (**e**). Value represents mean ± SEM of three replicates. * *p* < 0.05 vs. control. Actin was considered as the internal loading control. BEP treatment of C12A1 showed an enhancement of epithelial marker keratin 19 and reduction of mesenchymal marker vimentin transcript level as compared to control.

If cancer progresses to the metastatic phase, it will lead to poor outcomes. Cell migration and epithelial to mesenchymal transition (EMT) are crucial steps in the cascade of events leading up to metastatis. Thus, we wanted to know the effect of the BEP on cell migration and EMT of breast cancer MDA-MB-231 cells. We performed a scratch assay to determine the rate of cell migration. The data showed that the BEP extract significantly blocked cell migration (Figure 6a,b). Furthermore, BEP inhibited EMT of breast cancer cells, which was determined by an increased gene expression of the epithelial gene, keratin 19, and decreased gene expression of the mesenchymal gene, vimentin when compared to control (Figure 6c–e).

### 3.5. Influence of BEP on Stemness Property in MDA-MB-231 Cells

**Figure 7:**
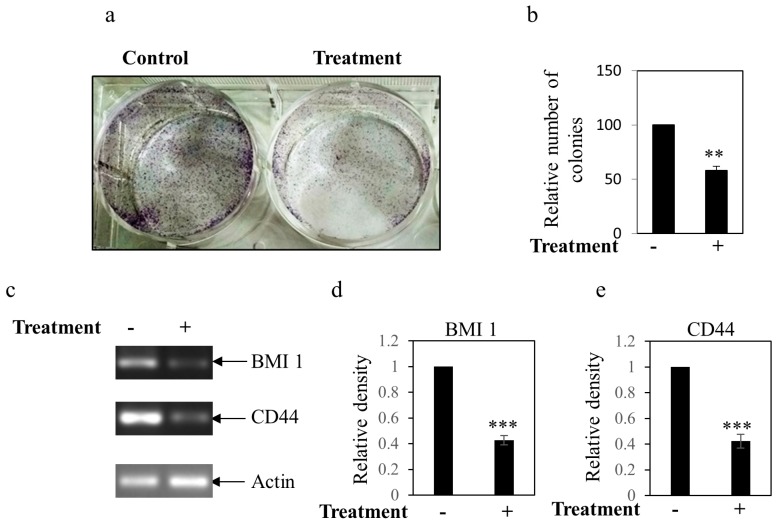
Effect of BEP on the stemness property of breast cancer cell (**a**) Colony formation of MDA-MB-231 cells after a consecutive 5 days of incubation with BEP treatment. Colonies were visualized by inverted bright field microscope. (**b**) Bars represent the number of colony count. Value represents the mean ± SEM of triplicate measurements, ** *p* < 0.001 vs. control. Treated cells decreased colony formation as compared to control. (**c**) Effect of BEP on the expression of the genes associated with the stemness of breast cancer cells from the total RNA extracted. Bars (**d**,**e**) showed the densitometry analysis (ratio of concerned gene/Actin) of the bands for BMI1 (**d**) and CD44 (**e**). Value represents mean ± SEM of three replicates. *** *p* < 0.001 vs. control. Actin was considered as the internal loading control. Transcript levels of cancer stemness-associated genes BMI1 and CD44 were downregulated in response to BEP.

Stemness is accountable for metastasis, drug resistance, and tumor recurrence. To determine the effect of the BEP on stemness property, we performed a colony formation assay, the results of which showed a fewer number of colonies in case of extract treated cells as compared to control (Figure 7a,b). Moreover, RT-PCR analysis showed inhibition of the expressions of cancer stemness-associated BMI1 and CD44 genes, markers of stemness, when cancer cells were treated with BEP as compared to control (Figure 7c–e).

## 4. Discussion

Moderately halophilic bacteria which grow at 0.5 to 2.5 M salinity are widely distributed in different saline habitats such as saline lakes, solar salterns, saline soils and salted foods [48]. During the last two decades, extensive studies on hypersaline environments in many geographical areas have been carried out and many moderately halophilic bacterial species have been classified according to their taxonomic and phylogenetic position [6]. Mostly 16S rRNA gene sequencing approach has been used in studying the distribution of microorganisms in such an environment [49]. In the present study, we report the presence of *Piscibacillus* sp., a moderately halo-alkaliphilic bacterium for the first time in India.

C12A1 showed 98.8% 16S rRNA gene sequence similarity with *Piscibacillus halophilus*, which was isolated from the water of the hypersaline lake Howz-Soltan, located near Qom city in central Iran [27] and 98.5% with *Piscibacillus salipiscarius* isolated from fermented fish (pla-ra) collected from a market in Ratchaburi Province, Thailand [50]. It is indicated that species of *Piscibacillus* are found in varied habitats such as saline-alkaline soil, fermented fish, among sea-anemones, and even in fish sauce. Two distinct sub-clades were visualized in the 16S rRNA gene phylogeny, which include different strains of both species mentioned above. Although C12A1 is phylogenetically closer to *P. salipiscarius*, it showed a distinct lineage without support of a significant bootstrap (<50) indicating its uncertain position in the tree (Figure 4). In the sub-clade which was represented by *P. salipiscarius*, some sequences are included that are designated to genus *Filobacillus*.

C12A1 has the potential to produce a yellow pigment, while bacterial colonies of earlier studied strains were white/creamy in color. The largest group of bacterial pigments belongs to the class of compounds known as carotenoids [51]. UV-visible absorption spectrum of pigments extracted from C12A1 also showed absorption peaks at 428 nm, 450 nm, and 480 nm indicating that carotenoids are the main components in the extract.

Both *Piscibacillus halophilus* and *Piscibacillus* sp. C12A1 were not able to produce acid from different carbohydrates (D-glucose, sucrose, D-fructose, glycerol); however, *Piscibacillus salipiscarius* was able to produce acid using these carbohydrate sources. Halophiles are potential source of various enzymes (lipase, protease, esterase, xylanase, and cholesterol oxidase) which are not only stable at different salt concentrations but may also be active at high temperature and pH values [52,53]. Qualitative analyses have shown that C12A1 was able to hydrolyze skimmed milk, gelatin, and Tween 80, which indicates its potential to produce industrially important protease and lipase enzyme.

A remarkable feature of C12A1 is the anticancer activity of BEP against metastatic breast cancer cells (MDA-MB-231). This is a novel finding; this type of activity has not been observed so far with other strains of *Piscibacillus*. In contrast with early stage cancer cell line MCF-7, our study herein focused on more aggressive late stage metastatic cancer cells MDA-MB-231. There was a clear anti-proliferative activity observed with a low inhibitory concentration (IC_50_) of 50% at 186.73 µg/mL. The BEP downregulated expression of the proliferative Bcl-xL and cell cycle promoting CDK2 gene (Figure 5). The BEP extract also blocked cell migration and EMT of MDA-MB-231 cells (Figure 6). Furthermore, the BEP seems to inhibit cancer stemness property since the treatment of MDA-MB-231 cells with BEP mitigated colony formation while it simultaneously inhibited stemness-related (CD44 and BMI-1) gene expressions (Figure 7). These results highly suggest that bioactive compounds found in BEP of C12A1, belonging to genus *Piscibacillus* could serve as potential therapeutic agents against breast cancer. However, further studies are required to identify the bioactive compounds present in the crude extract and to understand the underlying molecular mechanism(s) of the bioactive compound(s). A huge amount of moderately halophilic bacteria thrives in saline habitats which are inadequately explored for biotechnological applications. The present study provides primary evidence that halophilic bacterial strains are novel natural sources for the detection of novel bioactive substances.

## Figures and Tables

**Table 1:** Characteristics of the strain C12A1 and its differences with other phylogenetically related species *Piscibacillus salipiscarius* and *Piscibacillus halophilus.*

Characteristic	C12A1	*Piscibacillus salipiscarius* *	*Piscibacillus halophilus* **
Cell size (µm)	0.3–0.5 × 1.5–3.0	0.4–0.5 × 1.5–4.0	0.5–0.7 × 2.5–4.0
Gram reaction	+	+	+
Spore shape and position	Oval, terminal in swollen sporangia	Oval, terminal in swollen sporangia	Oval, terminal in swollen sporangia
Motility	+	+	+
NaCl range (optimum) (%, *w*/*v*)	5–25 (10–15)	2–30 (10–20)	1–20 (10)
Temperature range (optimum) (°C)	15–48 (37)	15–48 (37)	15–55 (35)
pH range (optimum)	6.0–10.0 (8.0)	5.0–9.0 (7.0)	7.0–10.0 (7.5)
Oxidase	+	+	+
Catalase	+	+	+
Hydrolysis of:			
Gelatin	+	+	+
Starch	−	+	−
Skimmed milk	+	+	+
Tween 80	+	−	+
Urea	−	−	ND
eduction of nitrate	+	−	−
Use of citrate	−	−	ND
Production of indole	−	−	−
Acid production from:			
D-glucose	−	+	−
D-maltose	−	−	−
D-Xylose	−	−	−
D-fructose	−	+	−
Glycerol	−	+	−
Sucrose	−	+	−
Pigment production	+	ND	ND
Antibiotic test:			
Tetracycline	S	ND	S
Kanamycin	R	ND	R
Gentamycin	S	ND	R

ND indicates no data available; S indicates sensitive; R indicates resistant; + indicates positive test; − indicates negative test; * Tanasupawat et al. 2007; ** Amoozegar et al. 2009.

## Supplementary Materials

The following are available online at http://www.mdpi.com/2076-2607/7/2/34/s1.
