# Supplementary material for: A Piscibacillus sp. Isolated from A Soda Lake Exhibits Anticancer Activity Against Breast Cancer MDA-MB-231 Cells
Source: Microorganisms. 2019 Jan 26;7(2):34. doi: 10.3390/microorganisms7020034 (PMC6406920; doi:10.3390/microorganisms7020034)
Supplement: Supplementary file 1 [file microorganisms-07-00034-s001.pdf]

Table S1: Sequence details of human gene specific primers used in PCR

Gene	Forward Primer	Reverse Primer
Bcl-xL	ACCCCAGGGACAGCATATCA	TGCGATCCGACTCACCAATA
CDK2	AGAAAATCCGCCTGGACACT	GAGAGCAGAGGCATCCATGA
CD44	CGGACACCATGGACAAGTTT	GAAAGCCTTGCAGAGGTCAG
BMI1	GTCCAAGTTCACAAGACCAGACC	ACAGTCATTGCTGCTGGGCATCG
Keratin 19	GCGGGACAAGATTCTTGGTG	CTTCAGGCCTTCGATCTGCAT
Vimentin	TCCAGCAGCTTCCTGTAGGT	CCCTCACCTGTGAAGTGGAT
Actin	AGCACTGTGTTGGCGTACAG	AGAGCTACGAGCTGCCTGAC
